# *trans*-Tetra­kis(1-allyl-1*H*-imidazole-κ*N* ^3^)bis­(thio­cyanato-κ*N*)nickel(II)

**DOI:** 10.1107/S1600536812001584

**Published:** 2012-01-21

**Authors:** Shao-Mei Zheng, Yan-Ling Jin

**Affiliations:** a College of Mechanical Engineering, Qingdao Technological University, Qingdao 266033, People’s Republic of China; b Key Laboratory of Advanced Materials, Qingdao University of Science and Technology, Qingdao 266042, People’s Republic of China

## Abstract

The structure of the title compound, [Ni(NCS)_2_(C_6_H_8_N_2_)_4_], consists of isolated mol­ecules of [Ni(NCS)_2_(Aim)_4_] (Aim = 1-allyl­imidazole), which contain a distorted octa­hedral NiN_6_ chromophore. The NCS^−^ anions are *trans* and four N atoms from the 1-allyl­imidazole ligands define the equatorial plane. The mean Mn—N(Aim) and Mn—N(NCS) distances are 2.105 (2) and 2.098 (2) Å, respectively. Weak C—H⋯N inter­actions contribute to the crystal packing stability.

## Related literature

In the corresponding nickel compound [Ni(NCS)_2_(1-methyl­imidazole)_4_] (Liu *et al.*, 2005 ▶), the Ni^II^ ions have a distorted octa­hedral environment.
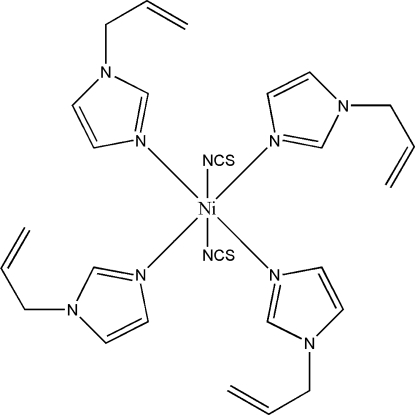


## Experimental

### 

#### Crystal data

[Ni(NCS)_2_(C_6_H_8_N_2_)_4_]
*M* *_r_* = 607.45
Triclinic, 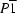

*a* = 8.8390 (18) Å
*b* = 9.5390 (19) Å
*c* = 10.515 (2) Å
α = 70.22 (3)°
β = 65.29 (3)°
γ = 86.66 (3)°
*V* = 754.3 (3) Å^3^
*Z* = 1
Mo *K*α radiation
μ = 0.82 mm^−1^
*T* = 293 K
0.20 × 0.10 × 0.10 mm

#### Data collection

Enraf–Nonius CAD-4 diffractometer
Absorption correction: ψ scan (North *et al.*, 1968 ▶) *T*_min_ = 0.854, *T*_max_ = 0.923
2934 measured reflections
2741 independent reflections
2367 reflections with *I* > 2σ(*I*)
*R*_int_ = 0.019
3 standard reflections every 200 reflections intensity decay: 1%

#### Refinement

*R*[*F* ^2^ > 2σ(*F* ^2^)] = 0.043
*wR*(*F* ^2^) = 0.124
*S* = 1.00
2741 reflections
178 parameters
H-atom parameters constrained
Δρ_max_ = 0.47 e Å^−3^
Δρ_min_ = −0.69 e Å^−3^

### 

Data collection: *CAD-4 EXPRESS* (Enraf–Nonius, 1989 ▶); cell refinement: *CAD-4 EXPRESS* (Enraf–Nonius, 1989 ▶); data reduction: *XCAD4* (Harms & Wocadlo, 1995 ▶); program(s) used to solve structure: *SHELXTL* (Sheldrick, 2008 ▶); program(s) used to refine structure: *SHELXTL* (Sheldrick, 2008 ▶); molecular graphics: *SHELXTL* (Sheldrick, 2008 ▶); software used to prepare material for publication: *SHELXTL* (Sheldrick, 2008 ▶) and local programs.

## Supplementary Material

Crystal structure: contains datablock(s) global, I. DOI: 10.1107/S1600536812001584/hg5160sup1.cif

Structure factors: contains datablock(s) I. DOI: 10.1107/S1600536812001584/hg5160Isup2.hkl

Supplementary material file. DOI: 10.1107/S1600536812001584/hg5160Isup3.cdx

Additional supplementary materials:  crystallographic information; 3D view; checkCIF report

## Figures and Tables

**Table:** 

Ni—N4	2.090 (2)
Ni—N5	2.098 (2)
Ni—N2	2.120 (2)
S—C13	1.631 (3)

**Table:** 

N4^i^—Ni—N5	90.41 (9)
N4—Ni—N5	89.59 (9)
N4^i^—Ni—N2	87.44 (9)
N4—Ni—N2	92.56 (9)
N5—C13—S	178.0 (3)

Symmetry code: (i) 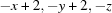.

**Table 2:** Hydrogen-bond geometry (Å, °)

*D*—H⋯*A*	*D*—H	H⋯*A*	*D*⋯*A*	*D*—H⋯*A*
C4—H4*A*⋯N5	0.93	2.74	3.139 (4)	107
C7—H7*A*⋯N3	0.93	2.54	2.862 (5)	101
C11—H11*A*⋯N5	0.93	2.87	3.187 (5)	102
C10—H10*A*⋯N5^i^	0.93	2.70	3.125 (4)	109
C5—H5*A*⋯N5^i^	0.93	2.69	3.134 (4)	110
C9—H9*A*⋯N5^ii^	0.97	2.97	3.793 (5)	143

Symmetry codes: (i) 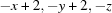; (ii) 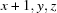.

**Fig. 1.**
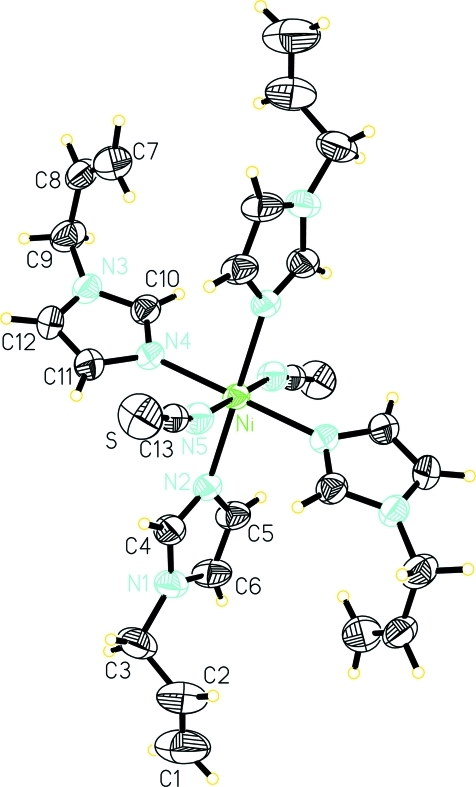
The structure of the title compound, showing 50% probability displacement ellipsoids and the atom-numbering scheme.

## supplementary crystallographic information

### Comment

The molecular structure of (I) is shown in Fig. 1. The Ni atom displays an octahedral coordination geometry, with six N atoms from two thiocyanate anions and four 1-allylimidazole ligands. The equatorial plane of the complex is formed by four Ni—*N*(1-allylimadazole) bonds with lengths of 2.090 (2) and 2.120 (2) Å, and the axial positions are occupied by two N-bonded NCS groups [Ni—N(NCS) = 2.098 (2) Å]. These values agree well with those observed in [Ni(NCS)_2_(1-methyl-1*H*-imidazole)_4_] (Liu *et al.*, 2005). The values of the bond angles around nickel atoms are close to those expected for a regular octahedral geometry, the N—Ni—N angles range from 87.44 (9) to 92.56 (9) °, and the thiocyanate ligands are almost linear. Weak C—H···N interactions contribute to the crystal packing stability.

In the corresponding nickel compound [Ni(NCS)_2_(1-methylimidazole)_4_] (Liu, *et al.*, 2005), the Ni^II^ ions have a distorted octahedral environment.

### Experimental

The title compound was prepared by the reaction of 1-allylimidazole (1.21 g, 20 mmol) with NiSO_4_.6H_2_O (1.31 g, 5 mmol) and potassium thiocyanate (0.98 g, 10 mmol) by means of hydrothermal synthesis in stainless-steel reactor with Teflon liner at 393 K for 24 h. Analysis, calculated for C_26_H_32_NiN_10_S_2_: C 51.41, H 5.31, N 23.06%; found: C 51.76, H 5.40, N 23.35%. Single crystals suitable for X-ray measurements were obtained by recrystallization from ethanol at room temperature.

### Refinement

H atoms were positioned geometrically(C—H = 0.93–0.97 Å) and allowed to ride on their parent atoms with *U*_iso_(H) = 1.2 times *U*_eq_(C).

### Crystal data

**Table:**

[Ni(NCS)_2_(C_6_H_8_N_2_)_4_]	*Z* = 1
*M**_r_* = 607.45	*F*(000) = 318
Triclinic, *P*1	*D*_x_ = 1.337 Mg m^−^^3^
Hall symbol: -P 1	Mo *K*α radiation, λ = 0.71073 Å
*a* = 8.8390 (18) Å	Cell parameters from 25 reflections
*b* = 9.5390 (19) Å	θ = 10–13°
*c* = 10.515 (2) Å	µ = 0.82 mm^−^^1^
α = 70.22 (3)°	*T* = 293 K
β = 65.29 (3)°	Block, green
γ = 86.66 (3)°	0.20 × 0.10 × 0.10 mm
*V* = 754.3 (3) Å^3^	

### Data collection

**Table:**

Enraf–Nonius CAD-4 diffractometer	2367 reflections with *I* > 2σ(*I*)
Radiation source: fine-focus sealed tube	*R*_int_ = 0.019
graphite	θ_max_ = 25.3°, θ_min_ = 2.3°
ω/2θ scans	*h* = 0→10
Absorption correction: ψ scan (North *et al.*, 1968)	*k* = −11→11
*T*_min_ = 0.854, *T*_max_ = 0.923	*l* = −11→12
2934 measured reflections	3 standard reflections every 200 reflections
2741 independent reflections	intensity decay: 1%

### Refinement

**Table:**

Refinement on *F*^2^	Primary atom site location: structure-invariant direct methods
Least-squares matrix: full	Secondary atom site location: difference Fourier map
*R*[*F*^2^ > 2σ(*F*^2^)] = 0.043	Hydrogen site location: inferred from neighbouring sites
*wR*(*F*^2^) = 0.124	H-atom parameters constrained
*S* = 1.00	*w* = 1/[σ^2^(*F*_o_^2^) + (0.092*P*)^2^] where *P* = (*F*_o_^2^ + 2*F*_c_^2^)/3
2741 reflections	(Δ/σ)_max_ < 0.001
178 parameters	Δρ_max_ = 0.47 e Å^−^^3^
0 restraints	Δρ_min_ = −0.68 e Å^−^^3^

### Special details

**Table:**

Geometry. All e.s.d.'s (except the e.s.d. in the dihedral angle between two l.s. planes) are estimated using the full covariance matrix. The cell e.s.d.'s are taken into account individually in the estimation of e.s.d.'s in distances, angles and torsion angles; correlations between e.s.d.'s in cell parameters are only used when they are defined by crystal symmetry. An approximate (isotropic) treatment of cell e.s.d.'s is used for estimating e.s.d.'s involving l.s. planes.
Refinement. Refinement of *F*^2^ against ALL reflections. The weighted *R*-factor *wR* and goodness of fit *S* are based on *F*^2^, conventional *R*-factors *R* are based on *F*, with *F* set to zero for negative *F*^2^. The threshold expression of *F*^2^ > σ(*F*^2^) is used only for calculating *R*-factors(gt) *etc*. and is not relevant to the choice of reflections for refinement. *R*-factors based on *F*^2^ are statistically about twice as large as those based on *F*, and *R*- factors based on ALL data will be even larger.

### Fractional atomic coordinates and isotropic or equivalent isotropic displacement parameters (Å^2^)

**Table:**

	*x*	*y*	*z*	*U*_iso_*/*U*_eq_	
Ni	1.0000	1.0000	0.0000	0.03248 (18)	
S	0.90268 (13)	0.62180 (10)	−0.17752 (10)	0.0667 (3)	
N1	0.8248 (3)	0.6671 (3)	0.4349 (3)	0.0495 (6)	
C1	0.5245 (7)	0.4590 (5)	0.7843 (5)	0.1058 (17)	
H1A	0.5948	0.4304	0.8324	0.127*	
H1B	0.4099	0.4556	0.8399	0.127*	
N2	0.9261 (3)	0.8588 (2)	0.2250 (2)	0.0391 (5)	
C2	0.5851 (6)	0.5028 (4)	0.6428 (4)	0.0805 (12)	
H2A	0.5100	0.5303	0.5996	0.097*	
N3	1.5200 (3)	0.9732 (3)	−0.1668 (3)	0.0455 (6)	
C3	0.7651 (5)	0.5141 (4)	0.5395 (4)	0.0699 (10)	
H3A	0.8308	0.4835	0.5970	0.084*	
H3B	0.7801	0.4470	0.4844	0.084*	
N4	1.2458 (3)	0.9421 (2)	−0.0627 (2)	0.0391 (5)	
C4	0.8908 (4)	0.7128 (3)	0.2850 (3)	0.0473 (7)	
H4A	0.9091	0.6492	0.2308	0.057*	
N5	0.9365 (3)	0.8207 (2)	−0.0471 (3)	0.0435 (5)	
C5	0.8802 (4)	0.9078 (3)	0.3426 (3)	0.0481 (7)	
H5A	0.8905	1.0073	0.3345	0.058*	
C6	0.8185 (4)	0.7922 (4)	0.4713 (3)	0.0553 (8)	
H6A	0.7790	0.7963	0.5668	0.066*	
C7	1.6338 (5)	1.1428 (4)	−0.4780 (4)	0.0670 (9)	
H7A	1.5227	1.1025	−0.4226	0.080*	
H7B	1.6737	1.1937	−0.5802	0.080*	
C8	1.7320 (4)	1.1281 (3)	−0.4132 (3)	0.0546 (8)	
H8A	1.8418	1.1705	−0.4737	0.066*	
C9	1.6888 (4)	1.0502 (4)	−0.2513 (4)	0.0582 (8)	
H9A	1.7686	0.9779	−0.2412	0.070*	
H9B	1.6990	1.1231	−0.2089	0.070*	
C10	1.3775 (3)	1.0383 (3)	−0.1235 (3)	0.0435 (6)	
H10A	1.3729	1.1394	−0.1351	0.052*	
C11	1.3071 (4)	0.8078 (3)	−0.0683 (3)	0.0506 (7)	
H11A	1.2426	0.7179	−0.0335	0.061*	
C12	1.4764 (4)	0.8268 (3)	−0.1325 (4)	0.0548 (8)	
H12A	1.5486	0.7537	−0.1497	0.066*	
C13	0.9239 (3)	0.7367 (3)	−0.0999 (3)	0.0368 (6)	

### Atomic displacement parameters (Å^2^)

**Table:**

	*U*^11^	*U*^22^	*U*^33^	*U*^12^	*U*^13^	*U*^23^
Ni	0.0336 (3)	0.0344 (3)	0.0275 (3)	0.00381 (18)	−0.00890 (19)	−0.01389 (19)
S	0.0922 (7)	0.0590 (5)	0.0670 (6)	0.0058 (5)	−0.0374 (5)	−0.0384 (4)
N1	0.0551 (14)	0.0469 (13)	0.0343 (12)	0.0015 (11)	−0.0123 (11)	−0.0075 (10)
C1	0.120 (4)	0.092 (3)	0.062 (3)	−0.026 (3)	−0.003 (3)	−0.015 (2)
N2	0.0394 (12)	0.0399 (12)	0.0322 (11)	0.0050 (9)	−0.0089 (9)	−0.0139 (9)
C2	0.082 (3)	0.078 (3)	0.060 (2)	−0.028 (2)	−0.020 (2)	−0.0060 (19)
N3	0.0347 (12)	0.0550 (14)	0.0427 (13)	0.0081 (10)	−0.0145 (10)	−0.0151 (11)
C3	0.086 (3)	0.0514 (19)	0.0476 (18)	−0.0001 (17)	−0.0167 (18)	−0.0012 (15)
N4	0.0374 (12)	0.0424 (12)	0.0344 (11)	0.0064 (10)	−0.0113 (10)	−0.0153 (9)
C4	0.0545 (17)	0.0448 (15)	0.0367 (14)	0.0073 (13)	−0.0128 (13)	−0.0159 (12)
N5	0.0460 (13)	0.0406 (12)	0.0423 (12)	0.0033 (10)	−0.0136 (10)	−0.0188 (10)
C5	0.0529 (17)	0.0489 (16)	0.0363 (14)	−0.0017 (13)	−0.0093 (12)	−0.0188 (13)
C6	0.065 (2)	0.0638 (19)	0.0296 (14)	−0.0056 (15)	−0.0101 (13)	−0.0176 (13)
C7	0.071 (2)	0.074 (2)	0.0478 (18)	0.0108 (18)	−0.0182 (17)	−0.0209 (17)
C8	0.0395 (15)	0.0573 (18)	0.0527 (18)	0.0033 (13)	−0.0047 (14)	−0.0207 (15)
C9	0.0373 (15)	0.077 (2)	0.0538 (18)	0.0022 (14)	−0.0162 (14)	−0.0186 (16)
C10	0.0420 (15)	0.0449 (15)	0.0415 (15)	0.0051 (12)	−0.0138 (12)	−0.0177 (12)
C11	0.0469 (16)	0.0398 (15)	0.0531 (17)	0.0062 (12)	−0.0123 (14)	−0.0139 (13)
C12	0.0484 (17)	0.0524 (18)	0.0534 (17)	0.0205 (14)	−0.0150 (14)	−0.0172 (14)
C13	0.0374 (13)	0.0339 (13)	0.0339 (13)	0.0021 (10)	−0.0112 (11)	−0.0105 (11)

### Geometric parameters (Å, °)

**Table:**

Ni—N4^i^	2.090 (2)	C3—H3A	0.9700
Ni—N4	2.090 (2)	C3—H3B	0.9700
Ni—N5	2.098 (2)	N4—C10	1.308 (3)
Ni—N5^i^	2.098 (2)	N4—C11	1.372 (3)
Ni—N2^i^	2.120 (2)	C4—H4A	0.9300
Ni—N2	2.120 (2)	N5—C13	1.152 (3)
S—C13	1.631 (3)	C5—C6	1.337 (4)
N1—C4	1.345 (4)	C5—H5A	0.9300
N1—C6	1.363 (4)	C6—H6A	0.9300
N1—C3	1.461 (4)	C7—C8	1.285 (5)
C1—C2	1.271 (5)	C7—H7A	0.9300
C1—H1A	0.9300	C7—H7B	0.9300
C1—H1B	0.9300	C8—C9	1.493 (4)
N2—C4	1.314 (3)	C8—H8A	0.9300
N2—C5	1.364 (3)	C9—H9A	0.9700
C2—C3	1.490 (6)	C9—H9B	0.9700
C2—H2A	0.9300	C10—H10A	0.9300
N3—C10	1.342 (3)	C11—C12	1.353 (4)
N3—C12	1.354 (4)	C11—H11A	0.9300
N3—C9	1.461 (4)	C12—H12A	0.9300
			
N4^i^—Ni—N4	180.000 (1)	C10—N4—C11	105.5 (2)
N4^i^—Ni—N5	90.41 (9)	C10—N4—Ni	124.18 (18)
N4—Ni—N5	89.59 (9)	C11—N4—Ni	129.86 (19)
N4^i^—Ni—N5^i^	89.59 (9)	N2—C4—N1	111.4 (3)
N4—Ni—N5^i^	90.41 (9)	N2—C4—H4A	124.3
N5—Ni—N5^i^	180.000 (1)	N1—C4—H4A	124.3
N4^i^—Ni—N2^i^	92.56 (9)	C13—N5—Ni	167.0 (2)
N4—Ni—N2^i^	87.44 (9)	C6—C5—N2	110.3 (3)
N5—Ni—N2^i^	90.56 (9)	C6—C5—H5A	124.9
N5^i^—Ni—N2^i^	89.44 (9)	N2—C5—H5A	124.9
N4^i^—Ni—N2	87.44 (9)	C5—C6—N1	106.5 (3)
N4—Ni—N2	92.56 (9)	C5—C6—H6A	126.8
N5—Ni—N2	89.44 (9)	N1—C6—H6A	126.8
N5^i^—Ni—N2	90.56 (9)	C8—C7—H7A	120.0
N2^i^—Ni—N2	180.0	C8—C7—H7B	120.0
C4—N1—C6	106.7 (2)	H7A—C7—H7B	120.0
C4—N1—C3	127.1 (3)	C7—C8—C9	127.0 (3)
C6—N1—C3	126.2 (3)	C7—C8—H8A	116.5
C2—C1—H1A	120.0	C9—C8—H8A	116.5
C2—C1—H1B	120.0	N3—C9—C8	113.2 (3)
H1A—C1—H1B	120.0	N3—C9—H9A	108.9
C4—N2—C5	105.2 (2)	C8—C9—H9A	108.9
C4—N2—Ni	129.36 (19)	N3—C9—H9B	108.9
C5—N2—Ni	124.73 (18)	C8—C9—H9B	108.9
C1—C2—C3	126.0 (5)	H9A—C9—H9B	107.8
C1—C2—H2A	117.0	N4—C10—N3	111.6 (2)
C3—C2—H2A	117.0	N4—C10—H10A	124.2
C10—N3—C12	107.0 (2)	N3—C10—H10A	124.2
C10—N3—C9	125.9 (3)	C12—C11—N4	109.2 (3)
C12—N3—C9	126.7 (3)	C12—C11—H11A	125.4
N1—C3—C2	111.1 (3)	N4—C11—H11A	125.4
N1—C3—H3A	109.4	C11—C12—N3	106.6 (3)
C2—C3—H3A	109.4	C11—C12—H12A	126.7
N1—C3—H3B	109.4	N3—C12—H12A	126.7
C2—C3—H3B	109.4	N5—C13—S	178.0 (3)
H3A—C3—H3B	108.0		
			
N4^i^—Ni—N2—C4	101.9 (3)	C3—N1—C4—N2	177.8 (3)
N4—Ni—N2—C4	−78.1 (3)	N4^i^—Ni—N5—C13	118.9 (10)
N5—Ni—N2—C4	11.5 (2)	N4—Ni—N5—C13	−61.1 (10)
N5^i^—Ni—N2—C4	−168.5 (2)	N2^i^—Ni—N5—C13	26.3 (10)
N4^i^—Ni—N2—C5	−67.1 (2)	N2—Ni—N5—C13	−153.7 (10)
N4—Ni—N2—C5	112.9 (2)	C4—N2—C5—C6	0.1 (4)
N5—Ni—N2—C5	−157.6 (2)	Ni—N2—C5—C6	171.3 (2)
N5^i^—Ni—N2—C5	22.4 (2)	N2—C5—C6—N1	−0.1 (4)
C4—N1—C3—C2	−120.9 (4)	C4—N1—C6—C5	0.2 (4)
C6—N1—C3—C2	56.7 (5)	C3—N1—C6—C5	−177.8 (3)
C1—C2—C3—N1	−121.4 (5)	C10—N3—C9—C8	−76.4 (4)
N5—Ni—N4—C10	148.6 (2)	C12—N3—C9—C8	95.5 (4)
N5^i^—Ni—N4—C10	−31.4 (2)	C7—C8—C9—N3	6.1 (5)
N2^i^—Ni—N4—C10	58.0 (2)	C11—N4—C10—N3	−0.5 (3)
N2—Ni—N4—C10	−122.0 (2)	Ni—N4—C10—N3	−173.39 (17)
N5—Ni—N4—C11	−22.5 (2)	C12—N3—C10—N4	0.4 (3)
N5^i^—Ni—N4—C11	157.5 (2)	C9—N3—C10—N4	173.7 (3)
N2^i^—Ni—N4—C11	−113.1 (2)	C10—N4—C11—C12	0.3 (3)
N2—Ni—N4—C11	66.9 (2)	Ni—N4—C11—C12	172.7 (2)
C5—N2—C4—N1	0.0 (3)	N4—C11—C12—N3	−0.1 (4)
Ni—N2—C4—N1	−170.67 (18)	C10—N3—C12—C11	−0.2 (3)
C6—N1—C4—N2	−0.1 (4)	C9—N3—C12—C11	−173.4 (3)

Symmetry codes: (i) −*x*+2, −*y*+2, −*z*.

### Hydrogen-bond geometry (Å, °)

**Table:**

*D*—H···*A*	*D*—H	H···*A*	*D*···*A*	*D*—H···*A*
C4—H4A···N5	0.93	2.74	3.139 (4)	107
C7—H7A···N3	0.93	2.54	2.862 (5)	101
C11—H11A···N5	0.93	2.87	3.187 (5)	102
C10—H10A···N5^i^	0.93	2.70	3.125 (4)	109
C5—H5A···N5^i^	0.93	2.69	3.134 (4)	110
C9—H9A···N5^ii^	0.97	2.97	3.793 (5)	143

Symmetry codes: (i) −*x*+2, −*y*+2, −*z*; (ii) *x*+1, *y*, *z*.
